# Stress Response During a Three-Month Follow-Up of a Historical Cohort of People with Cancer: Subjective Hyper-Energy and the DYMERS Framework for a Future Preventive Approach

**DOI:** 10.3390/jcm15166354

**Published:** 2026-08-17

**Authors:** Federica Sancassiani, Goce Kalcev, Elisa Pintus, Stefano Lorrai, Elisa Cantone, Clelia Madeddu, Olga Mulas, Giovanni Caocci, Sergio Machado, Marco Cruciata, Angela Muscettola, Rosangela Caruso, Maria Giulia Nanni, Martino Belvederi Murri, Luigi Grassi, Mauro Giovanni Carta

**Affiliations:** 1 Department of Medical Sciences and Public Health, University of Cagliari, 09042 Cagliari, Italy; 2 Unit of Psychosomatic Rehabilitation, AOU Cagliari, University Hospital of Cagliari “San Giovanni di Dio”, 09123 Cagliari, Italy; 3 Hematology Unit, “A. Businco” Hospital, 09121 Cagliari, Italy; 4 Center of Neuroscience, Neurodiversity Institute, Queimados 26325-010, Brazil; 5 Laboratory of Panic and Respiration, Institute of Psychiatry, Federal University of Rio de Janeiro (IPUB/UFRJ), Rio de Janeiro 22290-140, Brazil; 6 Department of Neuroscience and Rehabilitation, Institute of Psychiatry, University of Ferrara, 44121 Ferrara, Italy; 7 Hospital University Psychiatry Unit, “S. Anna” Hospital, Local Health Trust, 44124 Ferrara, Italy

**Keywords:** cancer, stress, hyper-energy, DYMERS, rhythm dysregulation

## Abstract

**Background:** This study examined whether baseline subjective hyper-energy was associated with depressive symptom changes over three months in people with cancer. **Methods:** This secondary exploratory analysis used a historical oncology cohort of 263 participants. Subjective hyper-energy was defined using SF-12 (Short Form Health Survey 12-item) item 10, and depressive symptoms were assessed with the PHQ-9 (Patient Health Questionnaire, 9-item) at baseline and follow-up. Inverse-probability weighting (IPW) examined attrition bias. **Results:** Follow-up PHQ-9 data were available for 112 participants (42.6%). At baseline, PHQ-9 scores were lower with than without subjective hyper-energy (4.27 ± 3.42 vs. 7.48 ± 4.39; *p* < 0.001). At follow-up, the difference was smaller and nonsignificant (5.61 ± 3.85 vs. 7.04 ± 3.71; *p* = 0.068). Unweighted scores increased by 1.33 ± 4.28 points with subjective hyper-energy and decreased by 0.44 ± 3.33 points without it (*p* = 0.020). After IPW, the between-group difference in change was attenuated to 0.99 points (95% CI −0.73 to 2.76). **Conclusions:** Baseline subjective hyper-energy was associated with fewer concurrent depressive symptoms. Although its association with subsequent change was attenuated after IPW, subjective hyper-energy may represent a potentially informative characteristic deserving further investigation within the Dysregulation of Mood, Energy, and Social Rhythms (DYMERS) framework. Prospective studies with validated multidimensional measures are needed to clarify its relationship with depressive worsening and rhythm dysregulation.

## 1. Background

Depression is one of the most frequent psychological complications experienced by people with cancer and is associated with poorer quality of life, reduced treatment adherence, and less favorable clinical outcomes [1,2,3]. While low energy is a well-recognized correlate of depressive states, considerably less attention has been devoted to understanding whether the opposite condition, namely the subjective perception of being “full of energy”, may also have clinical significance under conditions of prolonged stress. Sleep disturbances and alterations in biological and social rhythms are also common among patients with cancer and may contribute to psychological vulnerability [4,5,6]. More broadly, delays, advances and desynchronization of circadian rhythms have been implicated in the pathophysiology of depression and may also have implications for treatment response [7]. These clinical observations provide the rationale for exploring whether different patterns of subjective energy are associated with different responses to chronic oncological stress.

To clarify why hyper-energy may have different meanings under prolonged stress, its relationship with bipolar-spectrum concepts should be briefly considered. According to the neo-Kraepelinian approach, the spectrum of bipolar disorders includes most mood disorders and other disorders that often coexist with bipolar disorders [8,9]. Mania, even in its subthreshold form, would somehow precede most depressive episodes, like the ash that follows the fire of mania [10,11]. The official DSM-5 classification system of the American Psychiatric Association (APA) did not accept the neo-Kraepelinian approach and clearly distinguished bipolar disorders from depressive disorders [12]. One of the main points of disagreement between these two perspectives concerns the interpretation of the large number of “false-positive” cases identified by the screening instruments most commonly used in epidemiological studies of bipolar disorders [13].

According to those who followed the APA, the phenomenon was interpreted as the result of limited specificity of the screening instruments and as an exaggeration, given the importance of bipolar disorders [14,15]. On the contrary, from a neo-Kraepelinian perspective, it was noted that the individuals classified as “false positives” presented a profile of clinical and sociodemographic risk factors similar to that observed in bipolar disorders and that the disorders found in the “false positives” (i.e., anxiety disorders, personality disorders and abuse of medications) were not only frequently comorbid with bipolar disorders but, in this case, often began years before the onset of bipolar disorder [16].

Some recent contributions have led to the formulation of an alternative hypothesis, prompted by the observation that the experience of the COVID-19 pandemic and the associated stress were accompanied by profound dysregulation of biological and social rhythms, including the regulation of the sleep–wake cycle, largely attributable to prolonged lockdown measures and social restrictions [17]. It was observed that positivity on the Mood Disorder Questionnaire (MDQ), one of the most widely used screening instruments for bipolar-spectrum disorders, was associated with a marked impairment in quality of life, even in individuals without any psychiatric diagnosis and among older adults, despite the latter group having a relatively low probability of subsequently developing bipolar disorders [18].

These findings suggested that the subjective perception of hyper-energy, although often considered an adaptive characteristic, might, under specific circumstances, be associated with psychological suffering related to prolonged stress rather than with resilience. Specifically, it was hypothesized that persistent hyper-energy and hyperactivity, when coupled with dysregulation of biological rhythms, including alterations of the sleep–wake cycle, might represent a maladaptive response to chronic stress rather than an adaptive one [19]. Consistent with this evidence, genetic variants believed to be linked to bipolar disorders have also been identified frequently in elderly people with characteristics of hyperactivity, but who are perfectly adapted and without any psychiatric disorder [20,21]. Comparisons between MDQ screening and candidate genetic variants have suggested that these indicators may identify different components of bipolar-spectrum vulnerability [22]. Recent exploratory research has also examined the variable number tandem repeat polymorphism of PER3, a circadian gene involved in non-visual responses to light, in relation to age at onset and clinical characteristics of Bipolar I Disorder. Although the findings were mixed and require replication in larger samples, they further support investigation of the possible relationship between circadian genetic variability and the clinical expression of bipolar disorder [23].

It was therefore hypothesized that a condition of hyper-energy and hyperactivity could be found with a different and progressively decreasing adaptive meaning: a) in conditions in which the person had to overcome a difficult but manageable challenge (i.e., excellent athletes at a difficult event); b) in conditions of prolonged stress in which the person was unable to overcome a difficult obstacle and the hyperactivity continued like “the wheels of a car spinning desperately in a quagmire from which one cannot move forward” (i.e.,: in work-related stress); and c) in conditions of frank pathology in which hyperactivity is no longer aimed at overcoming the difficulties that (possibly) produced the crisis. The second step would be characteristic of the proposed “Dysregulation of Mood, Energy, and Social Rhythms Syndrome” (DYMERS) [19], and the third would be bipolar mania. Both conditions would test positive on screeners such as the MDQ [24].

DYMERS has been proposed as a potential vulnerability condition for bipolar disorders, but also for other stress-related psychopathological outcomes depending on individual vulnerability [25]. Importantly, in the present study DYMERS is considered as a conceptual framework for interpreting the observed findings rather than as a syndrome directly assessed in the study population. Cancer provides a clinically relevant setting in which to explore this possible distinction between adaptive and dysregulated activation.

Based on these observations, it appeared worthwhile to investigate cohorts exposed to severe stress, such as patients with chronic diseases, particularly cancer, and to examine the response to stress in relation to the core features of subjective hyper-energy and hyperactivity. This approach is further supported by evidence showing that a relevant set of genes implicated in the pathophysiology of bipolar disorder also contributes to the development of somatic and chronic conditions, which may partly account for the elevated comorbidity rates observed in this population [26]. Among chronic medical conditions, several neoplasms have been reported to share genetic and inflammatory pathways with bipolar disorder [27,28], making cancer particularly relevant for investigating the interplay between subjective hyper-energy, stress responses, and vulnerability to psychiatric comorbidity. Taken together, these observations suggest that cancer represents a clinically meaningful model in which to explore whether individual differences in subjective hyper-energy are associated with different psychological responses to prolonged stress.

Historical cohorts of patients with cancer [29,30,31,32] were not originally established to study subjective hyper-energy or DYMERS. However, it has recently been demonstrated that the item 10 of the widely used Short Form Health Survey 12-item version (SF-12), and the corresponding item of the similar SF-36, was associated with the MDQ total score, particularly with items assessing excessive energy and activity [33]. While this evidence does not replace structured clinical assessments or validated measures of hyperactivity and biological rhythms, it supports the rationale for using item 10 of the SF-12 as a proxy indicator of subjective hyper-energy in exploratory analyses such as the present one.

The main areas of evidence relevant to the present study are summarized in Table 1. Whereas previous psycho-oncology research has primarily investigated depression in relation to fatigue and reduced energy, the possible significance of subjective hyper-energy during prolonged cancer-related stress remains largely unexplored. The table also distinguishes this established evidence from the more recent and still-hypothesis-generating DYMERS framework.

Based on this framework, our exploratory working hypothesis was that participants reporting subjective hyper-energy at T0 would have lower concurrent depressive symptom levels but might show a greater increase in depressive symptoms over the three-month follow-up. Thus, we conducted a secondary exploratory analysis of a three-month follow-up of a historical cohort of patients with oncological diseases [29,30,31,32]. Specifically, participants were grouped according to subjective hyper-energy at baseline (independent variable) to examine whether changes in depressive symptoms over time (dependent variable) differed between the two groups. This exploratory analysis was undertaken within the conceptual framework of the previously proposed Dysregulation of Mood, Energy, and Social Rhythms Syndrome (DYMERS), which hypothesizes that hyper-energy may represent a vulnerability condition under prolonged stress [19].

## 2. Methods

### 2.1. Design and Study Samples

This study was a secondary, retrospective and exploratory analysis of longitudinal data collected within a historical cohort of people with cancer undergoing treatment [29,30,31,32]. The reporting of this observational analysis was guided by the Strengthening the Reporting of Observational Studies in Epidemiology (STROBE) statement [34]. The present study examined the frequency of depressive symptoms and the episodes of depression (dependent variable) in participants grouped according to the presence or absence of subjective hyper-energy at the initial assessment (independent variable). Subjective hyper-energy, depressive symptoms, and depressive episodes were evaluated at T0 and T1, three months later. Given the exploratory nature of this study and the measures available in the historical dataset [29,30,31,32], the findings should be considered hypothesis-generating and used to guide future prospective studies integrating evidence from bipolar-spectrum research, chronic stress, depressive trajectories, and rhythm dysregulation within a psycho-oncology framework supported by dedicated assessment tools.

### 2.2. Recruitment

As described extensively in previously published papers [29,30,31,32], recruitment was conducted between 2018 and 2020 from the Oncology Unit of the University Hospital of Cagliari, Italy, and the Haematology and Stem Cell Transplantation Centre, Azienda di Rilievo Nazionale ad Alta Specializzazione “Brotzu”, Cagliari, Italy. Participants were enrolled if they were aged ≥18 years, without limitation by sex, and with a histologically confirmed malignant cancer in active treatment. The cohort included 263 participants, with a mean age of 61.21 ± 13.62 years; 132 (50.2%) were women. Of the total sample, 201 participants (76.4%) had solid tumors and 62 (23.6%) had hematological malignancies. The most frequently recorded solid tumor sites were gastroenteric (n = 91), breast (n = 32), gynecological (n = 32), urogenital (n = 17), and lung (n = 16). Twenty-nine participants (11.0%) had stage 1–2 disease and 234 (89.0%) had stage 3–4 disease. At baseline, nine participants (3.4%) were attending their first oncology visit, 102 (38.8%) had been receiving care for less than 6 months, 48 (18.3%) for 6–12 months, and 103 (39.2%) for more than 12 months; this information was missing for one participant (0.4%). Treatment intent was palliative for 149 participants (56.7%), adjuvant for 48 (18.3%), curative for 32 (12.2%), maintenance for 25 (9.5%), supportive for five (1.9%), and neoadjuvant for four (1.5%).

### 2.3. Study Tools

Perceived energy or vitality was assessed using item 10 of the Italian version of the SF-12 [35,36]: “How much of the time during the past 4 weeks did you have a lot of energy?” The Italian version of the SF-12 has demonstrated satisfactory reliability and construct validity for the assessment of physical and mental health-related quality of life [36]. In this exploratory analysis, responses of 5 (“almost always”) or 6 (“always”) were operationally labeled “subjective hyper-energy”. It was found that SF-12 item 10 was associated with MDQ scores, particularly with excessive energy and activity, and showed fair discrimination of MDQ positivity [33].

The self-administered scale of nine items, “Patient Health Questionnaire” (PHQ-9) [37] in the Italian version [38], was adopted for measuring depressive episodes and depressive symptoms. The overall score of PHQ-9 is the sum of the scores of the nine items, each asking about the core symptoms of a depressive episode according to the Diagnostic and Statistical Manual of Mental Disorders (DSM-5) criteria [12]. The PHQ-9 is a widely validated measure of depressive symptom severity, with satisfactory reliability, construct and criterion validity, and responsiveness to change, including evidence from oncology populations [39]. In this specific work, we took the 9/10 level as the cut-off, considering a score >9 as indicative of the presence of a depressive episode.

### 2.4. Ethical Aspect

The study protocol received approval from the Ethical Committee of Azienda Ospedaliero-Universitaria di Cagliari, Italy, in 2018 (number PG/2018/13269). Written informed consent, after being presented with comprehensive explanations of the objectives and methodologies of the study, informed on data security, and assured of their right to discontinue their participation at any point, was requested from each participant. All procedures were conducted according to the Helsinki Declaration [40].

### 2.5. Statistical Analysis

The comparison at T0 and T1 (three months later) was conducted between those who did not have subjective hyper-energy at T0 (who did not answer “5” or “6” to item 10 of the SF-12) and those who showed subjective hyper-energy at T0 (responding “5” or “6” to item 10 of the SF-12).

The comparison between means and standard deviations of numerical data between the two cohorts at T0 (PHQ-9 score and socio-demographic characteristics) and at the end of the follow-up (PHQ-9 score) was carried out using one-way ANOVA, as was the comparisons of changes in PHQ-9 scores from T0 to T1 between groups. Nominal variables were compared between the two cohorts at baseline (T0), including participants’ characteristics, the prevalence of depressive episodes at T0 and T1, and the incidence of new depressive episodes between T0 and T1. Differences between groups were assessed using the Chi-square test (with Yates’ continuity correction when appropriate) or Fisher’s exact test, depending on the expected cell frequencies.

To assess potential attrition bias, baseline characteristics of participants with and without an observed PHQ-9 score at T1 were compared using one-way ANOVA for continuous variables and Chi-square or Fisher’s exact tests for categorical variables. Absolute standardized mean differences (SMDs) were used to quantify imbalance, with values ≥0.10 considered potentially relevant.

Potential item-content overlap was examined by repeating the group comparisons using modified PHQ-9 scores excluding item 4 (fatigue/low energy) and, in a second analysis, both item 4 and item 3 (sleep disturbance). These scores were used only for sensitivity analyses, without applying diagnostic cut-offs.

Attrition bias was further assessed using inverse-probability-of-observation weighting (IPW), defining outcome observation as availability of the PHQ-9 total score at T1. Observation probabilities were estimated through multivariable logistic regression including age, sex, cancer type, cancer stage, timing of care, SF-12 item 10 score and baseline PHQ-9 score. One participant with an observed T1 outcome was excluded because baseline timing-of-care data were missing.

Stabilized weights were calculated using the overall probability of outcome observation as the numerator. Covariate balance after weighting was assessed using absolute SMDs. Weighted between-group differences were estimated for PHQ-9 at T1 and for change from T0 to T1, with differences calculated as the hyper-energy group minus the group without hyper-energy. Robustness was examined by truncating weights at the 1st and 99th percentiles. Standard errors and 95% confidence intervals were estimated by nonparametric bootstrap, refitting the observation model in each replicate; 980 of 1000 replicates converged and were included.

## 3. Results

**Figure 1:**
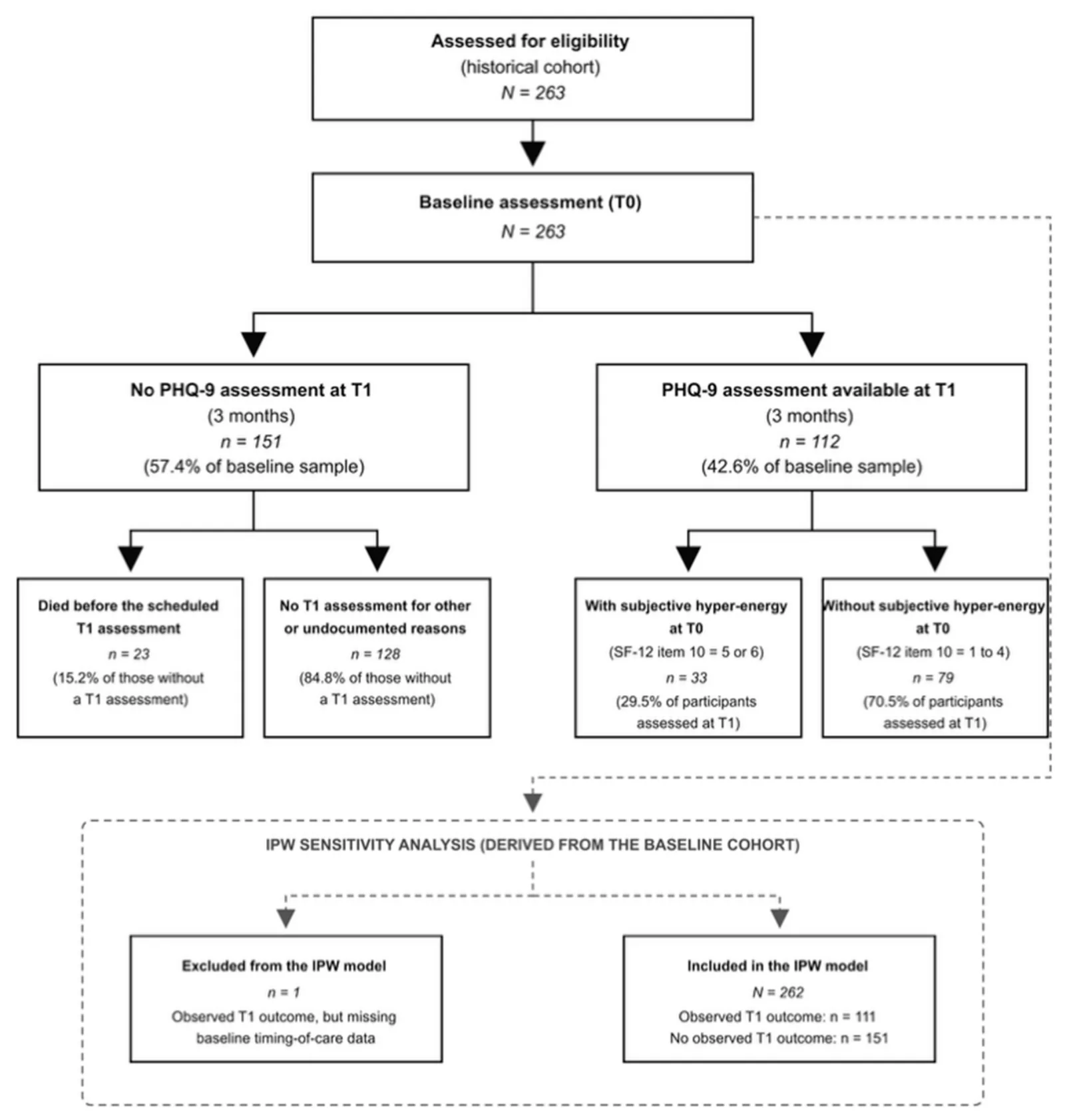
The flow of participants through the study.

Table 2 illustrates the characteristics of the study sample, and Figure 1 shows the flow of participants through the study. After three months of follow-up, 112 participants from the initial sample had an available PHQ-9 assessment at T1 (42.6%), whereas 151 (57.4%) did not. Of the 151 participants without a PHQ-9 assessment at T1, 23 (15.2%) had died before the scheduled follow-up assessment, whereas the remaining 128 had no T1 assessment for other or undocumented reasons. Among the 112 participants assessed at T1, 33 (29.5%) had hyper-energy at T0, defined as an SF-12 item 10 score of 5 or 6, whereas 79 (70.5%) did not, defined as an SF-12 item 10 score between 1 and 4. One participant with an observed T1 outcome had missing baseline timing-of-care data and was therefore excluded from the propensity model. The IPW analysis consequently included 262 participants, of whom 111 had an observed T1 outcome.

The detailed comparison of participants with and without an observed T1 outcome is reported in Supplementary Table S1. Absolute standardized mean differences indicated that the largest baseline imbalances concerned cancer type (absolute SMD = 0.44) and depressive episodes at T0 (absolute SMD = 0.34). Smaller imbalances were observed for age (absolute SMD = 0.15) and sex (absolute SMD = 0.12), whereas the remaining characteristics showed absolute SMDs below 0.10. In the adjusted observation model, participants with a hematological rather than solid cancer were less likely to have an observed T1 outcome (adjusted OR 0.27, 95% CI 0.13–0.58; *p* = 0.001). Higher baseline PHQ-9 scores were also associated with a lower probability of outcome observation (adjusted OR per five-point increase 0.68, 95% CI 0.49–0.94; *p* = 0.019) (Supplementary Table S2).

Within the sample assessed at T1, sex and age did not differ significantly between participants with and without hyper-energy. Women were numerically less frequent in the hyper-energy group than in the group without hyper-energy, although this difference did not reach statistical significance (39.4% vs. 59.5%; χ^2^ = 3.781, *p* = 0.052). Participants who were at their first visit or had received cancer care for less than six months were less frequent in the hyper-energy group than in the group without hyper-energy (30.3% vs. 50.6%; χ^2^ = 3.89, *p* = 0.049). However, no significant differences were found between the groups in cancer stage or cancer type.

At the beginning of the observation, participants with hyper-energy had lower PHQ-9 scores than participants without hyper-energy (4.27 ± 3.42 vs. 7.48 ± 4.39; F (1, 110) = 14.046, *p* < 0.001). At T1, the difference was smaller and did not reach statistical significance (5.61 ± 3.85 vs. 7.04 ± 3.71; F (1, 110) = 3.394, *p* = 0.068). In the unweighted analysis, the hyper-energy group showed a greater increase in depressive symptoms over three months than the group without hyper-energy: an increase of 1.33 ± 4.28 points compared with a decrease of 0.44 ± 3.33 points, respectively (F (1, 110) = 5.577, *p* = 0.020) (Table 3).

Sensitivity analyses excluding the PHQ-9 fatigue/low-energy item confirmed both the baseline difference between groups (*p* = 0.002) and the unweighted difference in change (*p* = 0.033). When both the fatigue and sleep items were excluded, the baseline difference persisted (*p* = 0.002), whereas the difference in change was attenuated to borderline statistical significance (*p* = 0.050) (Supplementary Table S4).

Stabilized inverse-probability-of-observation weights had a mean of 1.00 and a maximum of 3.97. After weighting, all absolute standardized mean differences were below 0.10, indicating adequate balance on the observed baseline covariates (Supplementary Table S3, Panel A). In the complete-covariate analytic sample, the unweighted PHQ-9 T1 difference between participants with and without hyper-energy was −1.26 points (95% CI −2.66 to 0.18). After IPW, the corresponding difference was −2.34 points (95% CI −3.99 to −0.75), with lower scores in the hyper-energy group. For the change from T0 to T1, the unweighted between-group difference was 1.85 points (95% CI 0.19 to 3.40), whereas the IPW difference was attenuated to 0.99 points (95% CI −0.73 to 2.76). Results were similar after truncating weights at the 1st and 99th percentiles. Thus, the finding of a greater increase in depressive symptoms among participants with hyper-energy was sensitive to adjustment for attrition (Supplementary Table S3, Panel B).

A similar pattern was observed for depressive episodes, defined as PHQ-9 scores >9. At baseline, depressive episodes were less frequent among participants with hyper-energy than among those without hyper-energy (9.1% vs. 26.6%; χ^2^ = 4.230, *p* = 0.040). At T1, the corresponding proportions were 18.2% and 22.8%, respectively, and the between-group difference was no longer statistically significant (χ^2^ = 0.293, *p* = 0.588) (Table 4). Thus, descriptively, the frequency increased from T0 to T1 in the hyper-energy group and decreased slightly in the group without hyper-energy. Among participants without a depressive episode at baseline, new depressive episodes occurred in 5 of 30 participants with hyper-energy and 4 of 58 participants without hyper-energy (two-sided Fisher’s exact *p* = 0.264; RR 2.42, 95% CI 0.70–8.34).

## 4. Discussion

Our study found that people with cancer undergoing treatment who perceive themselves as always or almost always full of energy, at the beginning of the observation, appear to have fewer depressive symptoms and a lower frequency of depressive episodes than those who do not feel full of energy. This result is expected, given that fatigue and a low energy level are the main symptoms of the depressive episode, and it is consistent with the clinical observation that fatigue and low energy are among the most common and burdensome symptoms in oncology, often associated with poorer mood outcomes [4]. Over the three-month follow-up, however, depressive symptoms increased in the group with baseline subjective hyper-energy and decreased slightly in the group without subjective hyper-energy, while the baseline difference in screen-positive depressive episodes was no longer evident at T1.

These descriptive findings were qualified by the attrition sensitivity analysis. Inverse-probability weighting attenuated the between-group difference in PHQ-9 change, whereas the weighted T1 comparison continued to indicate lower depressive symptom levels among participants with baseline subjective hyper-energy. Therefore, although the unweighted findings suggest a possible relative worsening of depressive symptoms in the hyper-energy group, the present data do not provide robust evidence that hyper-energy predicts a greater increase in depressive symptoms.

The concept of a “Dysregulation of Mood, Energy, and Rhythms Syndrome” (DYMERS) may nevertheless provide a hypothesis-generating framework for interpreting changes in energy and mood under prolonged oncological stress. Longitudinal studies in cancer cohorts have identified subgroups of patients with worsening depressive trajectories during continuing disease- and treatment-related stress [41], while sleep disturbance and rest–activity rhythm disruption have emerged as potentially relevant pathways [5,42]. Our findings suggest that baseline hyper-energy may identify a clinically distinct subgroup, but they cannot establish that hyper-energy changes from a protective characteristic into a marker of vulnerability.

Several studies in different languages [43,44] have found that the PHQ-9’s optimal cut-off scores ranged from >5 to ≥15. However, if the use of a high cut-off (i.e., 15/16) guarantees the screening of few false positives but relatively many false negatives, the choice of a mean-low cut-off, such as 9/10, while guaranteeing few false negatives, leads to a high percentage of people who, despite achieving a high score, would not be diagnosed as cases of major depressive disorder on a clinical level. The problem is what those forms are on a clinical level that, despite involving depressive symptoms and impairment, do not reach the threshold for a diagnosis of a major depressive episode (in which the symptoms must be present not only for at least 15 days but all day for most of the day). This issue is particularly relevant in oncology, where patients often present with subthreshold depressive symptoms related to chronic illness and treatment burden, which may not reach the threshold for major depressive disorder but still cause significant impairment [45].

It is known that, with a low PHQ-9 cut-off, these cases are in 30% to 60% of people identified as positive [43,44]. In our study, we talk about people subjected to continuous stress because they suffer from a chronic pathology. Likely, the diagnosis of adjustment disorder could therefore apply here. In cancer populations, adjustment disorder is frequently diagnosed when mood and stress-related symptoms emerge in close connection with disease course and treatment, yet its validity and reproducibility remain debated [3]. The diagnosis of adjustment disorder appears more like a basket that collects everything that cannot be put elsewhere than a diagnosis following current systems [46].

In fact, on the one hand, it is in contradiction to the anti-theoretical approach of current psychiatric classification (as DSM and ICD) because an etiological determinant is required in DSM-5; a “marked distress out of proportion to the severity or intensity of the stressor...” [12]. Thus, it is the individual vulnerability that causes the “abnormal” reaction. How difficult it is for this diagnosis to be reproducible in a system that, otherwise, is only strictly descriptive. This is indicated by the fact that from 1988 to 1997 (the time of the diffusion of selective serotonin reuptake inhibitor [SSRI] antidepressants), the diagnosis of adjustment disorder with depressed mood decreased from 28% to 14.7% in liaison psychiatry units of United States general hospitals. In the same period, the diagnosis of major depressive disorder with concomitant medical illness (which is the theme of this paper) increased from 6.4% to 14.7% in the same hospital units [47].

In other words, these difficulties outline a “no man’s land” of chronic stress reactions that needs to be better characterized at a descriptive syndromic level, together with the clinical features potentially associated with vulnerability. Within this framework, baseline subjective hyper-energy may represent a potentially informative but nonspecific clinical characteristic.

A transdiagnostic interpretation of these findings is therefore warranted. Subjective hyper-energy is not specific to a single disorder or mechanism and may reflect several overlapping processes, including individual coping responses, anxiety or heightened arousal, sleep disturbance, treatment- or medication-related effects, physical symptom burden, and differences in cancer severity or progression. The present secondary analysis could not disentangle these possible contributions. Moreover, because the longitudinal group difference was attenuated after adjustment for attrition, the study cannot establish that hyper-energy independently predicts subsequent depressive worsening or represents a marker of vulnerability under chronic stress. Its possible relationship with stress response and rhythm dysregulation should consequently be regarded as a hypothesis requiring confirmation.

### 4.1. Strengths and Limitations

The limitations are that our study is based on a cohort built with different objectives, and in which the present hypotheses were tested with tools not specifically developed for this purpose. This was, therefore, a secondary, exploratory analysis. In particular, hyper-energy was defined using a single SF-12 item rather than a validated multidimensional measure of energy, activation or biological rhythms. The construct examined here should consequently not be equated with hyperactivity, hypomania or a validated DYMERS diagnosis. Furthermore, the PHQ-9 total score includes items assessing fatigue or low energy and sleep disturbance. Therefore, the baseline association with SF-12 item 10 may partly reflect overlapping item content rather than a distinct protective effect of subjective hyper-energy.

Attrition was substantial: only 112 of the 263 baseline participants had an available PHQ-9 assessment at T1. Among the 151 participants without a T1 assessment, 23 had died before the scheduled follow-up. Participants with hematological cancer and those with higher baseline PHQ-9 scores were less likely to have an observed follow-up outcome, indicating that attrition was related to measured baseline characteristics. Inverse-probability weighting achieved good balance on the included covariates, but the weighted analysis attenuated the difference in PHQ-9 change between the hyper-energy and non-hyper-energy groups. The longitudinal finding is therefore sensitive to assumptions regarding attrition. Furthermore, IPW can address selection associated with observed variables but cannot eliminate bias due to unmeasured predictors of follow-up or to outcomes that remained systematically missing after conditioning on the measured covariates.

For the 23 participants who died before T1, the follow-up PHQ-9 outcome was intrinsically unavailable; therefore, inverse-probability weighting cannot entirely eliminate the potential selection introduced by mortality.

The primary longitudinal findings were based on unadjusted group comparisons, and no multivariable repeated-measures or mixed-effects model was fitted. Therefore, baseline differences, particularly in PHQ-9 scores, and residual confounding may partly account for the observed between-group changes. The analysis could not account for potentially relevant clinical factors, including specific cancer treatments, pain, corticosteroid or psychotropic medication use, cancer-related fatigue, and inflammatory burden; residual confounding by these factors therefore cannot be excluded. These findings should consequently be interpreted as descriptive and hypothesis-generating rather than as evidence of an independent longitudinal association. The hyper-energy group was also relatively small, resulting in limited precision, particularly for estimates of incident depressive episodes. Moreover, the PHQ-9 is a screening instrument and does not establish a clinical diagnosis; depressive episodes were not confirmed through structured diagnostic interviews. The observational secondary design does not permit causal inference regarding whether hyper-energy contributes to subsequent depressive symptoms. The short follow-up period, the exploratory nature of the analyses and the number of statistical comparisons further limit confirmatory interpretations.

Finally, the secondary nature of the study, the substantial loss to follow-up and the recruitment of a heterogeneous historical cohort from specific oncology services limit the generalizability of the findings. In particular, the results may not be applicable to patients treated in other clinical settings or phases of cancer care, or to patients with more severe clinical conditions who were unable to complete the follow-up assessment.

Despite these limitations, the contribution seems to be of interest and suggests the conduct of studies specifically oriented to the evaluation of chronic stress and the impact of hyper-energy on the dysregulation of rhythms, such as sleep–wake and rest–activity rhythms, which can clarify the temporal sequence of the onset of symptoms. This work’s main strength is its contribution to this area of research, acknowledging that its ideas possess only heuristic value. Future studies should incorporate validated measures of hyper-energy and stress response to test the proposed hypothesis more rigorously. Such studies should also include repeated assessments of mood and sleep using dedicated validated instruments, such as the Insomnia Severity Index, together with sleep diaries, validated chronotype questionnaires, and objective assessments of rest–activity rhythms using actigraphy, as well as a longer follow-up period to clarify the temporal sequence of changes in energy and depressive symptoms [48,49].

Indeed, it should be emphasized that, if our findings are confirmed, they would hold significant importance for preventive measures in the oncology field. However, the present results do not support interventions aimed specifically at suppressing or directly modifying hyper-energy. Rather, future research should examine whether interventions that promote adaptive stress regulation and regular sleep–wake and activity rhythms help prevent mood deterioration in potentially vulnerable patients. Studies conducted by our group suggest that physical exercise [50,51] and/or the use of virtual reality-based cognitive remediation may be particularly promising in this regard [52,53,54].

### 4.2. Novelties and Contributions

Previous psycho-oncology research has predominantly examined depression in relation to fatigue, reduced energy and sleep disturbance. The present study extends this perspective by exploring subjective hyper-energy, rather than low energy, in patients undergoing cancer treatment. It also applies the DYMERS conceptual framework to an oncological cohort and examines the short-term evolution of depressive symptoms according to baseline subjective energy levels. Unlike previous DYMERS studies conducted in other stress-related contexts, this analysis evaluates hyper-energy during the prolonged stress associated with cancer care. The findings do not validate DYMERS or establish hyper-energy as an independent predictor of depressive worsening; rather, the study’s contribution is to identify a relatively unexplored clinical characteristic and generate hypotheses for prospective research using validated measures of energy, stress response, sleep and biological rhythms.

### 4.3. Clinical Implications

The clinical implications of this study should be interpreted cautiously. Rather than supporting immediate changes to clinical practice, the findings suggest that future oncological and psycho-oncological research should examine whether assessing not only depressive symptoms but also patients’ subjective energy levels provides clinically useful information about possible emotional and rhythm dysregulation. Individuals presenting with hyper-energy at the beginning of treatment may initially appear resilient and less vulnerable to mood symptoms, but the present findings do not demonstrate that this profile masks an underlying dysregulation or independently predisposes patients to depressive reactions under chronic stress. Routine monitoring of energy, regularity of rhythms, and mood fluctuations throughout treatment should therefore be evaluated prospectively to determine whether it can facilitate early identification of at-risk cancer patients and allow timely preventive interventions. The present study was not designed to develop or validate a clinical prediction model; accordingly, it does not establish baseline subjective hyper-energy as a clinical marker, risk-classification tool or preventive target. This caution is particularly important because subjective hyper-energy was operationalized using a single SF-12 item and sleep–wake or rest–activity rhythms were not directly assessed.

From a practical standpoint, if these findings are confirmed, future psycho-oncological programs could evaluate strategies aimed at promoting adaptive regulation of energy and stabilizing biological rhythms. Potential approaches for evaluation could include tailored psychoeducation on stress and rest–activity balance, behavioral interventions promoting regular sleep and eating hygiene and structured physical activity, and virtual-reality-based cognitive remediation and relaxing approaches to support adaptive regulation. These interventions require formal evaluation and should not currently be recommended specifically for the purpose of suppressing or directly modifying hyper-energy. Clinicians should also be aware of the diagnostic ambiguity surrounding subthreshold or adjustment-related depressive symptoms in oncology, ensuring that emotional distress is addressed even when it does not meet the formal criteria for major depressive disorder.

Ultimately, future studies should determine whether incorporating rhythm- and energy-focused assessment and intervention into routine oncological care may contribute to more personalized psychosocial support and improve emotional outcomes. Any possible role in preventing clinically significant depressive symptoms will require confirmation in appropriately designed prospective intervention studies.

## 5. Conclusions

In conclusion, baseline subjective hyper-energy was associated with fewer depressive symptoms at T0 among patients undergoing cancer treatment. Although the unweighted analysis suggested a greater subsequent increase in depressive symptoms in this group, this difference was attenuated after inverse-probability weighting for attrition. Hyper-energy may therefore represent a potentially informative clinical characteristic, but its role as a marker of vulnerability to depressive worsening remains uncertain. Its possible relationship with chronic stress and rhythm dysregulation should be investigated in larger, prospectively designed longitudinal studies using validated multidimensional measures.

## Figures and Tables

**Table 1:** Comparative overview of existing evidence and the incremental contribution of the present study.

Evidence Area	Existing Evidence	Principal Unresolved Issue	Incremental Contribution of the Present Study
Depression and cancer-related fatigue	Depression and fatigue or reduced energy frequently co-occur in patients with cancer and are associated with poorer quality of life and clinical outcomes [1,2,3,4,5,29].	Energy has predominantly been examined in terms of fatigue or reduced energy; subjective hyper-energy has received little attention.	Examines the opposite end of the subjective-energy dimension by comparing patients with and without baseline hyper-energy.
Bipolar-spectrum characteristics and cancer	Epidemiological and clinical studies have suggested associations between bipolar disorder or bipolar-spectrum traits and some oncological conditions, potentially involving shared biological and inflammatory pathways [26,27,28].	These studies do not establish whether subjective hyper-energy among patients without diagnosed bipolar disorder is associated with subsequent depressive symptoms during cancer treatment.	Explores subjective hyper-energy without equating it with bipolar disorder, hypomania or a bipolar-spectrum diagnosis.
DYMERS and stress-related hyperactivation	Previous DYMERS work proposed that persistent hyperactivation combined with disrupted biological and social rhythms may represent a maladaptive response to prolonged stress [18,19,24,25]. SF-12 item 10 has also been associated with MDQ scores in non-oncological samples [33].	DYMERS remains a hypothesis-generating framework and has not been validated as a clinical syndrome; evidence in oncology is particularly limited.	Extends the DYMERS hypothesis to an oncological context using SF-12 item 10 as a proxy for subjective hyper-energy, without claiming to assess or validate DYMERS.
Present study	Secondary exploratory analysis of a historical cohort of patients receiving cancer care, with baseline hyper-energy and PHQ-9 assessed over three months.	The design does not directly assess hyperactivity, bipolarity, chronic stress or biological rhythms and cannot establish causal mechanisms.	Provides preliminary longitudinal evidence on the relationship between baseline subjective hyper-energy and depressive symptoms and identifies priorities for specifically designed prospective studies.

Note: DYMERS, Dysregulation of Mood, Energy, and Social Rhythms Syndrome; MDQ, Mood Disorder Questionnaire; PHQ-9, Patient Health Questionnaire-9; and SF-12, 12-item Short Form Health Survey.

**Table 2:** Characteristics of the study sample.

	Overall Sample (n = 263)	Not Retained at T1 (n = 151)	Retained at T1 (n = 112)	Retained at T1 Without Hyper-Energy at T0 (n = 79)	Retained at T1 with Hyper-Energy at T0 (n = 33)	Retained vs. Not Retained Among Participants Without Hyper-Energy at T0	Retained vs. Not Retained Among Participants with Hyper-Energy at T0	Hyper-Energy vs. No Hyper-Energy Among Participants Retained at T1
**Sex** **(female)**	132 (50.2%)	72 (47.7%)	60 (53.6%)	47 (59.5%)	13 (39.4%)	χ^2^ = 1.876 *p* = 0.171	χ^2^ = 0.010 *p* = 0.919	χ^2^ = 3.781 *p* = 0.052
**Age**	61.21 ± 13.62	60.32 ± 14.88	62.39 ± 11.68	62.19 ± 12.14	62.88 ± 10.67	F = 0.505 *p* = 0.4782 df 1; 197	F = 1.760 *p* = 0.1894 df 1; 63	F = 0.090 *p* = 0.765 df 1; 110
**First visit or time since the beginning of care <6 months**	111 (42.2%)	61 (40.4%)	50 (44.6%)	40 (50.6%)	10 (30.3%)	χ^2^ = 1.172 *p* = 0.190	χ^2^ = 0.376 *p* = 0.540	χ^2^ = 3.89 *p* = 0.049
**Cancer type (blood)**	62 (23.6%)	47 (31.1%)	15 (13.4%)	10 (12.7%)	5 (15.1%)	χ^2^ = 6.96 *p* = 0.008	χ^2^ = 5.26 *p* = 0.0218	Fisher’s exact test: *p* = 0.76
**Cancer type (solid)**	201 (76.4%)	104 (68.9%)	97 (86.6%)	69 (87.3%)	28 (84.8%)	χ^2^ = 6.96 *p* = 0.008	χ^2^ = 5.26 *p* = 0.0218	Fisher’s exact test: *p* = 0.76
**Cancer stage** **(1 or 2)**	29 (11.0%)	18 (11.9%)	11 (9.8%)	9 (11.4%)	2 (6.1%)	χ^2^ = 0.018 *p* = 0.893	Fisher’s exact test *p* = 0.427	Fisher’s exact test: *p* = 0.721
**Cancer stage** **(3 or 4)**	234 (89.0%)	133 (88.1%)	101 (90.2%)	70 (88.6%)	31 (93.9%)	χ^2^ = 0.018 *p* = 0.893	Fisher’s exact test *p* = 0.427	Fisher’s exact test: *p* = 0.721
**Depressive** **Episodes** **(PHQ-9 score >9)**	79 (30%)	55 (36.4%)	24 (21.4%)	21 (26.6%)	3 (9.1%)	χ^2^ = 4.425 *p* = 0.0354	χ^2^ = 0.590 *p* = 0.442	χ^2^ = 4.230 *p* = 0.0397

**Table 3:** Evolution of the PHQ-9 score during the observation time in people with and without hyper-energy.

	PHQ-9 Score (T0)	PHQ-9 Score (T1)	Difference T1 − T0
**With** **hyper-energy** **(n = 33)**	4.27 ± 3.42	5.61 ± 3.85	1.33 ± 4.28
**Without** **hyper-energy** **(n = 79)**	7.48 ± 4.39	7.04 ± 3.71	−0.44 ± 3.33
**ANOVA one-way**	F = 14.046 *p* < 0.001 1;110 df	F = 3.394 *p* = 0.068 1;110 df	F = 5.577 *p* = 0.020 1;110 df

**Table 4:** Depressive episodes score during the observation time in people with and without hyper-energy.

	Depressive Episodes PHQ-9 >9 (T0)	Depressive Episodes PHQ-9 >9 (T1)	New Cases with a Depressive Episode
**With** **hyper-energy** **(n = 33)**	3 (9.1%)	6 (18.2%)	5/30
**Without** **hyper-energy** **(n = 79)**	21 (26.6%)	18 (22.8%)	4/58
	χ^2^ = 4.229 *p* = 0.040	χ^2^ = 0.293 *p* = 0.588	Risk Ratio 2.42, 95% CI 0.70–8.34; Fisher’s exact *p* = 0.264

## Data Availability

Individual-level participant data are not publicly available because of the conditions of the original informed consent and ethical approval and the potential risk of re-identification. Anonymized aggregated data and the statistical analysis code supporting the reported results may be made available from the corresponding author upon reasonable and methodologically justified request. Requests will be evaluated in accordance with applicable institutional, ethical and data-protection requirements.

## Supplementary Materials

The following supporting information can be downloaded at: https://www.mdpi.com/article/10.3390/jcm15166354/s1, Table S1: Comparison of baseline characteristics between participants retained and not retained at T1; Table S2: Multivariable predictors of PHQ-9 outcome observation at T1; Table S3: Inverse-probability-weighted sensitivity analyses; Table S4: Sensitivity analyses using modified PHQ-9 scores excluding potentially overlapping somatic items.

## Abbreviations

DYMERS: Dysregulation of Mood, Energy, and Social Rhythms Syndrome; DSM-5: Diagnostic and statistical manual of mental disorders (latest edition); ICD: The International Classification of Diseases; APA: American Psychiatric Association; MDQ: Mood Disorder Questionnaire; SF-12: Short Form Health Survey 12; PHQ-9: Patient Health Questionnaire.
