# Supplementary material for: Stress Response During a Three-Month Follow-Up of a Historical Cohort of People with Cancer: Subjective Hyper-Energy and the DYMERS Framework for a Future Preventive Approach
Source: J Clin Med. 2026 Aug 17;15(16):6354. doi: 10.3390/jcm15166354 (PMC13513895; doi:10.3390/jcm15166354)
Supplement: Supplementary file 1 [file jcm-15-06354-s001.zip › jcm-4440262-supplementary.pdf]

SUPPLEMENTARY MATERIAL

Supplementary Table S1. Comparison of baseline characteristics between participants retained and not retained at T1

	**Overall sample (n=263)**	**Not retained at T1 (n=151)**	**Retained at T1 (n=112)**	**Retained at T1 vs. Not retained at T1**	**Absolute SMD**
**Sex (female)**	132 (50.2%)	72 (47.7%)	60 (53.6%)	χ^2=0.892 p=0.345	0.12
**Age**	61.21±13.62	60.32±14.88	62.39±11.68	F=1.485 p=0.224	0.15
**First visit or time since the beginning of care <6 months**	111 (42.2%)	61 (40.4%)	50 (44.6%)	χ^2=0.475 p=0.491	0.09
**Cancer type (blood)**	62 (23.6%)	47 (31.1%)	15 (13.4%)	χ^2=11.223 p<0.001	0.44
**Cancer type (solid)**	201 (76.4%)	104 (68.9%)	97 (86.6%)	χ^2=11.223 p<0.001	0.44
**Cancer stage (1 or 2)**	29 (11.0%)	18 (11.9%)	11 (9.8%)	χ^2=0.289 p=0.591	0.07
**Cancer stage (3 or 4)**	234 (89.0%)	133 (88.1%)	101 (90.2%)	χ^2=0.289 p=0.591	0.07
**Depressive Episodes at T0 (PHQ-9 score>9)**	79 (30%)	55 (36.4%)	24 (21.4%)	χ^2=6.880 p=0.009	0.34

Absolute SMD, absolute standardized mean difference. Values ≥0.10 were considered indicative of potentially relevant imbalance.

Attrition and inverse-probability-weighted analyses

Supplementary Table S2. Multivariable predictors of PHQ-9 outcome observation at T1

Predictor	Adjusted OR	95% CI	p-value	Scaling or reference category
Age (per 10 years)	1.08	0.88 to 1.34	0.454	Per 10-year increase
Female vs male	1.44	0.83 to 2.50	0.198	Male
Blood vs solid cancer	0.27	0.13 to 0.58	0.001	Solid cancer
Stage 3–4 vs 1–2	1.11	0.46 to 2.66	0.811	Stage 1–2
Care 6–12 vs <6 months	0.79	0.38 to 1.66	0.540	<6 months
Care >12 vs <6 months	1.51	0.79 to 2.90	0.213	<6 months
First visit vs <6 months	2.06	0.49 to 8.61	0.324	<6 months
Hyper-energy T0 (per point)	1.04	0.82 to 1.32	0.750	Per 1-point increase
PHQ-9 T0 (per 5 points)	0.68	0.49 to 0.94	0.019	Per 5-point increase

Note. The dependent variable was availability of the PHQ-9 total score at T1 (observed vs missing). The model included 262 participants: 111 with an observed outcome and 151 with a missing outcome. One participant with an observed T1 outcome was excluded because timing of care at baseline was missing. OR>1 indicates a higher probability of outcome observation. OR, odds ratio; CI, confidence interval; PHQ-9, Patient Health Questionnaire-9.

Supplementary Table S3. Inverse-probability-weighted sensitivity analyses

Panel A. Absolute standardized mean differences before and after IPW

Baseline covariate	Before IPW: observed vs missing	After IPW: weighted observed vs analytic cohort	Balance criterion
Age	0.16	0.05	<0.10
Female	0.13	0.02	<0.10
Blood cancer	0.46	0.02	<0.10
Stage 3–4	0.06	0.07	<0.10
Care 6–12 months	0.09	0.07	<0.10
Care >12 months	0.02	0.06	<0.10
First visit	0.10	0.02	<0.10
Hyper-energy T0	0.20	0.03	<0.10
PHQ-9 T0	0.32	0.02	<0.10

Note. Absolute SMDs are reported. Before weighting, SMDs compare participants with observed and missing PHQ-9 T1 outcomes in the complete-covariate analytic sample. After weighting, SMDs compare the weighted observed-outcome sample with the full analytic cohort. Values <0.10 indicate adequate balance by convention. Stabilized weights had a mean of 1.00, a maximum of 3.97, and an effective sample size of 91.1. SMD, standardized mean difference; IPW, inverse-probability weighting.

Panel B. Complete-case and IPW estimates of PHQ-9 group differences

Outcome	Analysis	Hyper-energy mean	No hyper-energy mean	Mean difference	95% CI
PHQ-9 T1	Complete-case	5.78	7.04	-1.26	-2.66 to 0.18
PHQ-9 T1	Stabilized IPW	5.45	7.79	-2.34	-3.99 to -0.75
PHQ-9 T1	IPW, weights truncated	5.45	7.67	-2.22	-3.89 to -0.65
PHQ-9 change T1−T0	Complete-case	1.41	-0.44	1.85	0.19 to 3.40
PHQ-9 change T1−T0	Stabilized IPW	0.41	-0.58	0.99	-0.73 to 2.76
PHQ-9 change T1−T0	IPW, weights truncated	0.41	-0.61	1.02	-0.70 to 2.76

Note. Mean differences are calculated as hyper-energy minus no hyper-energy. Negative PHQ-9 T1 differences indicate lower T1 scores in the hyper-energy group; positive change-score differences indicate a greater increase in the hyper-energy group. The complete-case rows refer to the complete-covariate analytic sample with 111 observed outcomes (32 with hyper-energy and 79 without hyper-energy); one additional participant with hyper-energy and an observed T1 outcome was excluded because baseline timing of care was missing. Stabilized IPW estimates were derived from the observation model in Supplementary Table S2. Truncated analyses limited weights to the 1st and 99th percentiles. Standard errors and 95% CIs were obtained by nonparametric bootstrap with the observation model refitted in each replicate (980 valid of 1,000 replicates). CI, confidence interval; IPW, inverse-probability weighting; PHQ-9, Patient Health Questionnaire-9.

Supplementary Table S4. Sensitivity analyses using modified PHQ-9 scores excluding potentially overlapping somatic items

Panel A. Modified PHQ-9 score excluding item 4 (fatigue/low energy; theoretical range 0–24)

Assessment	Subjective hyper-energy (n=33), mean±SD	No subjective hyper-energy (n=79), mean±SD	Mean difference (95% CI)[1]	F (df)	p-value
T0	3.42±3.02	5.81±3.92	−2.39 (−3.90 to −0.88)	9.794 (1,110)	0.0022
T1	4.36±3.27	5.35±3.19	−0.99 (−2.31 to 0.33)	2.217 (1,110)	0.1394
**Change (T1−T0)**	0.94±3.61	−0.46±2.88	1.40 (0.12 to 2.67)	4.686 (1,110)	0.0326

Panel B. Modified PHQ-9 score excluding item 4 (fatigue/low energy) and item 3 (sleep disturbance; theoretical range 0–21)

Assessment	Subjective hyper-energy (n=33), mean±SD	No subjective hyper-energy (n=79), mean±SD	Mean difference (95% CI)[1]	F (df)	p-value
T0	2.55±2.59	4.52±3.22	−1.97 (−3.23 to −0.72)	9.740 (1,110)	0.0023
T1	3.12±2.71	3.96±2.84	−0.84 (−1.99 to 0.31)	2.094 (1,110)	0.1507
**Change (T1−T0)**	0.58±3.36	−0.56±2.47	1.13 (0.00 to 2.27)[2]	3.913 (1,110)	0.0504

Notes. Values are unweighted complete-case estimates. Modified scores were calculated by subtracting the specified item(s) from the PHQ-9 total score at the corresponding assessment and were used only for sensitivity analyses; diagnostic cut-offs were not applied. Positive change scores indicate worsening depressive symptoms. Group comparisons were performed using one-way ANOVA. [1] Mean difference calculated as subjective hyper-energy minus no subjective hyper-energy. [2] The unrounded lower confidence limit was −0.002. CI, confidence interval; PHQ-9, Patient Health Questionnaire-9; SD, standard deviation; T0, baseline; T1, three-month follow-up.
